# Fatigue and Sleep-Disordered Breathing in Multiple Sclerosis: A Clinically Relevant Association?

**DOI:** 10.1155/2013/286581

**Published:** 2013-10-22

**Authors:** Ulf Kallweit, Christian R. Baumann, Michael Harzheim, Hildegard Hidalgo, Dieter Pöhlau, Claudio L. Bassetti, Michael Linnebank, Philipp O. Valko

**Affiliations:** ^1^Department of Neurology, University Hospital Zurich, Frauenklinikstraße 26, 8091 Zurich, Switzerland; ^2^Department of Neurology, Kamillus-Klinik, Hospitalstrasse 6, 53567 Asbach, Germany; ^3^Department of Neurology, University of Bern, Inselspital, Freiburgstrasse 10, 3010 Bern, Switzerland

## Abstract

*Background*. Fatigue in patients with multiple sclerosis (MS) is highly prevalent and severely impacts quality of life. Recent studies suggested that sleep-disordered breathing (SDB) significantly contributes to fatigue in MS. *Study Objective*. To evaluate the importance of routine respirography in MS patients with severe fatigue and to explore the effects of treatment with continuous positive airway pressure (CPAP). *Patients and Methods*. We prospectively assessed the presence of severe fatigue, as defined by a score of ≥5.0 on the Fatigue Severity Scale (FSS), in 258 consecutive MS patients. Ninety-seven patients (38%) suffered from severe fatigue, whereof 69 underwent overnight respirography. *Results*. We diagnosed SDB in 28 patients (41%). Male sex was the only independent associate of SDB severity (*P* = 0.003). CPAP therapy in 6 patients was associated with a significant reduction of FSS scores (5.8 ± 0.5 versus 4.8 ± 0.6, *P* = 0.04), but the scores remained pathological (≥4.0) in all patients. *Conclusion*. Respirography in MS patients with severe fatigue should be considered in daily medical practice, because SDB frequency is high and CPAP therapy reduces fatigue severity. However, future work is needed to understand the real impact of CPAP therapy on quality of life in this patient group.

## 1. Introduction

 Although fatigue has been increasingly recognized over the past two decades as one of the most frequent and most debilitating symptoms in patients with MS, there are still no insights into its neurobiological mechanisms, and current treatment options are highly frustrating [1–4]. In clinical practice, MS patients complaining about fatigue are usually first scrutinized for additional and potentially treatable comorbidities, such as depression, pain, anemia, or sleep-wake disturbances [5]. If there is no such cause of fatigue, the patient is considered to suffer from “MS-related fatigue,” that is, a disease-inherent symptom related to the underlying neuroimmunological and neurodegenerative processes, and off-label symptomatic treatment with stimulants of the central nervous system may be recommended [3].

 Recently, the need to search for sleep-wake disorders in MS patients has been reemphasized, as several groups observed a significant correlation with fatigue [6–10]. Specifically, sleep-disordered breathing (SDB) has been proposed as a potential risk factor for fatigue in MS. In the last year, a cross-sectional study in 48 MS patients suggested a predisposition for SDB [11], and two studies found that severe fatigue in MS was significantly associated with SDB and respiratory-related arousals [9, 12]. Furthermore, Côté et al. conducted the first controlled, nonrandomized clinical treatment study and reported a significant improvement of fatigue following treatment of SDB and other sleep disorders [13]. Very recently, Veauthier et al. were able to identify treatment of sleep disorders in MS patients as independent predictor of fatigue reduction [14].

 Taken together, there is increasing amount of data suggesting that SDB should be routinely screened for in all MS patients with severe fatigue. We therefore prospectively assessed the presence and severity of fatigue in consecutive, unselected MS patients seen in a tertiary center and suggested overnight respirography to MS patients with severe fatigue. Patients with SDB were offered CPAP therapy. The main purpose was to elucidate whether routine respirography can be recommended as a screening method in MS patients with severe fatigue and whether long-term CPAP therapy leads to a clinically meaningful improvement of fatigue. In addition, we aimed at examining potential predictive factors of SDB in MS patients with fatigue.

## 2. Patients and Methods

This prospective study was conducted at the Neurological Department of the Kamillus-Klinik in Asbach, Germany, from October 2010 until March 2011. The study protocol has been reviewed and approved by the local Institutional Review Boards, and all patients gave written informed consent before inclusion. 

### 2.1. Subjects and Clinical Assessment

 During the above-mentioned period, we prospectively evaluated 258 consecutive patients with multiple sclerosis (MS). The diagnosis of MS was definite in each patient and was made according to standard criteria [15]. The majority of patients suffered from secondary progressive MS (87%) (SPMS), and 13% had a relapsing remitting (RRMS) form. We measured fatigue severity with the Fatigue Severity Scale (FSS), a self-administered questionnaire with nine items which has been validated for various neurological disorders including MS [16, 17]. A final score greater than 4.0 is widely accepted to indicate the presence of fatigue. For the purpose of our study, we included only patients with severe fatigue defined by a FSS score ≥5.0. The rationale for including only patients with severe fatigue is given by the observation that beneficial treatment effects of CPAP therapy are usually most pronounced in patients with high levels of fatigue [18]. We identified 97 MS patients with severe fatigue. Sixty-nine of them agreed to undergo overnight respirography. We used the Epworth Sleepiness Scale (ESS) to assess sleepiness, with scores ≥10 indicating excessive daytime sleepiness (EDS) [19, 20]. Information on disease duration was taken from the patients' medical history. We estimated the severity of disease disability using the Kurtzke Expanded Disability Status Scale (EDSS) [21].

### 2.2. Sleep Studies

 Overnight (from 10 p.m. to 6 a.m.) respirography was performed using a portable device (EasyScreen 4.0, Respironixs, Germany), which measured nasal airflow, pulse rate, arterial oxygen saturation (finger pulse oximetry), and thoracic and abdominal movements. Respirography has been validated previously in patients with ischemic stroke [22]. Apnea was defined by a cessation of nasal airflow ≥10 seconds and hypopnea by a reduction of nasal airflow by ≥50% or ≥30%, when associated with an oxygen desaturation ≥4% [23]. Apnea-hypopnea index (AHI) was defined by the mean number of apneas and hypopneas per hour. SDB was diagnosed in patients with an AHI ≥ 5/h. SDB was classified as mild (AHI 5–15/h), moderate (AHI 15–30/h), and severe (AHI ≥ 30/h). Apneas were differentiated in obstructive, mixed, and central apneas according to standard criteria [23]. Apneas were obstructive if accompanied with continuous respiratory effort, central if unaccompanied by evidence of respiratory effort, and mixed if a central apnea developed respiratory effort with evidence of obstruction later in the apneic interval. A diagnosis of central sleep apnea was made in patients, in whom >50% of all respiratory events were central. In addition, calculation of the oxygen desaturation index (ODI) was performed. The magnitude of the oxygen desaturation considered for ODI was ≥4%. The analysis was made automatically and corrected visually. Titration of CPAP was performed during overnight video-polysomnography. The optimal therapeutic pressure was determined to eliminate snoring, apneic events with arousals, and oxyhemoglobin desaturations in all body positions and sleep stages. Patients received conventional CPAP therapy (REMstar M series, Philips Respironics or SOMNOcomfort2, Weinmann). Reevaluation of FSS and ESS scores was scheduled after a 6-month period of CPAP therapy, although significant short-term relief of both fatigue and sleepiness can be expected already after 3–6 weeks of CPAP therapy [18]. 

### 2.3. Statistics

 Continuous data were expressed as means and standard deviation (SD) and categorical variables as numbers and percentage. All analyses were performed with the Statistical Package for the Social Sciences (SPSS, version 17.0). For univariate analysis we used Student's *t*-test (for numerical scale variables) and **χ** ^2^-test (for nominal scale variables). To identify predictors of SDB severity, we performed stepwise multiple linear regression analysis for multivariate analysis, with AHI as dependent variable and the following independent variables: age, sex, disease duration, disease subtype, EDSS, BMI, FSS, and ESS scores. Correlation analyses were performed using the Spearman's coefficient. Significance was accepted at *P* < 0.05. 

## 3. Results

### 3.1. Demographic and Clinical Characteristics

 Sixty-nine MS patients with severe fatigue consented to overnight respirography. The majority were female (70%); mean age was 49.8 ± 9.2 years (range: 21–75 years). BMI was 26.0 ± 4.9 (range: 17–40). Most patients were severely affected by the disease, as reflected by a mean EDSS of 5.8 ± 1.4 (range: 1.0–8.5) and mean disease duration of 13.7 ± 8.8 years (range: 1–33 years). The patient group that declined respirography (*n* = 28) showed similar demographic and clinical characteristics (data not shown).

### 3.2. Sleep-Disordered Breathing

Overnight respirography revealed a mean AHI of 9.3 ± 16.9/h and a mean ODI of 10.5 ± 14.7%. SDB was found in 28 MS patients (41%) and was mild in 18 patients (26%), moderate in 3 patients (4%), and severe in 7 patients (10%). SDB was obstructive in all but one patient. MS patients with SDB were significantly older (*P* = 0.01) and tended to be more often male (*P* = 0.06). Likewise, AHI correlated with age (rho = 0.33, *P* = 0.006) but not with EDSS, disease duration, or BMI.  Table 1 provides the remaining demographic and clinical characteristics (Table 1).

### 3.3. Excessive Daytime Sleepiness

 The overall prevalence of excessive daytime sleepiness in our patient group was high (51%). The presence of excessive daytime sleepiness, however, was similar between patients with and without SDB (61% versus 44%, *P* = 0.13). Even when comparing patients with moderate-severe SDB and those without SDB, we did not detect any differences in the prevalence of SDB (70% versus 44%, *P* = 0.30). Similarly, there was no correlation between ESS scores and AHI (*r* = −0.05, *P* = 0.66).

### 3.4. Sex-Related Differences

**Figure 1:**
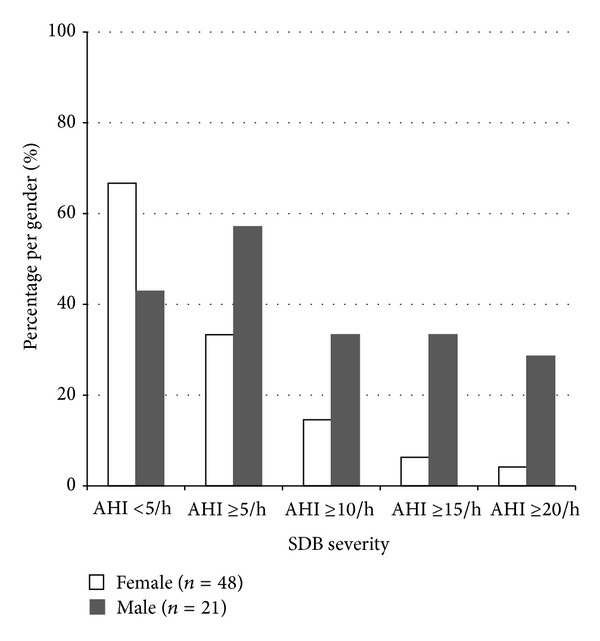
Male MS patients with severe fatigue were more frequently affected by SDB than female MS patients with severe fatigue.

 The gender ratio (f : m) was 3.6 : 1 in patients without SDB, but the male proportion significantly increased with more severe SDB (*P* = 0.005): the ratio was 2.6 : 1 in mild SDB, 0.5 : 1 in moderate SDB, and 0.4 : 1 in severe SDB. BMI was similar in both male and female patients (25.3 ± 3.8 versus 26.3 ± 5.2, *P* = 0.12), but AHI was significantly higher in male patients (18.5 ± 24.7 versus 5.2 ± 9.9, *P* < 0.001). Multiple regression analysis confirmed that male sex was an independent associate of SDB severity (*P* = 0.003).  Figure 1 shows the different relative distribution of SDB in male and female patients. Otherwise, the clinical characteristics were similar in both sex groups (Table 2).

### 3.5. Evolution of FSS and ESS under CPAP Therapy

**Figure 2:**
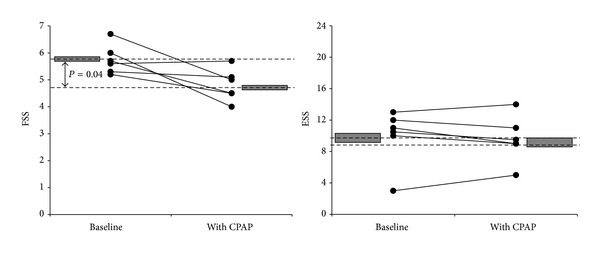
Follow-up under long-term treatment with continuous positive air pressure (CPAP) reveals significant reduction of FSS scores but not of ESS scores.

 We recommended CPAP therapy to all patients with an AHI ≥ 10/h (*n* = 14). Nine patients agreed, but 3 of them did not tolerate the device. The remaining 6 patients (2 with RRMS, 4 with SPMS) revealed a CPAP daily average adherence of ≥5 hours per night during the 6-month treatment. In these patients, AHI decreased during CPAP therapy from 39.2 ± 26.3/h to 5.2 ± 3.6/h (*P* = 0.02), and the minimal SaO_2_ improved from 71.5 ± 11.7% to 88.2 ± 1.9% (*P* = 0.02). EDSS remained stable (6.3 ± 1.3 versus 6.3 ± 1.2). At followup after 6 months, CPAP therapy was associated with a significant decrease of FSS scores (5.8 ± 0.5 versus 4.8 ± 0.6, *P* = 0.04), whereas ESS scores did not change (9.8 ± 3.5 versus 9.5 ± 3.0, *P* = 0.61) (Figure 2). However, despite the significant reduction of fatigue severity during CPAP therapy, FSS scores remained pathologic (≥4.0) in all treated patients.

## 4. Discussion

 Considering the increasing interest in SDB as a potential risk factor for fatigue in MS, the primary goal of this prospective investigation—so far the largest respirography study on SDB in MS—was to understand whether routine overnight respirography should be done in all MS patients with severe fatigue. We found a SDB frequency of 41% among MS patients with severe fatigue and demonstrated that CPAP therapy was associated with a significant improvement of fatigue severity, while sleepiness remained unchanged. Furthermore, only male sex could be identified as an sindependent predictor of SDB in MS patients with severe fatigue. Taken together, these findings indicate that routine respirography should be considered in this specific patient group, but future work is needed to confirm our results and establish the exact benefit of SDB treatment also in terms of quality of life.

 Although the observed SDB frequency was rather high, our study was not designed to estimate the prevalence of SDB in MS or to compare its prevalence between MS patients with and without fatigue, as we did not perform respirography in MS patients with FSS scores <5.0 and did not include a control group. Moreover, comparison to other studies is hampered by disparities of included patient cohorts, methodological and technical differences, and heterogeneous definitions of SDB. In this line, two recent studies on SDB and fatigue in MS differed with regard to the applied polysomnographic scoring criteria: one group used the Rechtschaffen and Kales criteria and the other study scored according to the 1999 American Academy of Sleep Medicine Task Force [9, 12, 24]. The two methods use different placement of EEG electrodes, which may affect the scoring of respiratory arousals. Thus, several reasons may account for the large discrepancy of reported prevalence of SDB in MS patients with or without fatigue. For instance, two earlier studies did not find any cases with SDB, as defined by an AHI ≥ 5/h, among 37 and 10 MS patients, respectively, although the majority of these patients suffered from fatigue [25, 26]. On the other hand, Kaminska et al. performed polysomnography in 37 MS patients with severe fatigue, as defined by the same criteria as in our study, and found severe SDB (AHI > 30/h) in 32%, relative to 8% among 25 MS patients without severe fatigue [12]. Using another fatigue questionnaire, Veauthier et al. reported a SDB prevalence of 27% and 2.5% in 26 MS patients with fatigue and 40 MS patients without fatigue, respectively [9]. Finally, Braley et al. reported a mean AHI of 17.0 ± 18.8/h in 48 MS patients, a much higher number than in our study [11]. Overall, these studies illustrate our uncertainty regarding prevalence and severity of SDB in MS and its relationship to fatigue. 

 Six MS patients with SDB and severe fatigue showed significant reduction of fatigue severity after 6 months of CPAP therapy: FSS scores decreased from 5.8 ± 0.5 to 4.8 ± 0.6. In other words, long-term CPAP therapy resulted in a 17% fatigue reduction. Our results are in line with the recent study of Côté et al. who treated 17 MS patients with SDB and fatigue, reporting a decrease of FSS scores from 5.0 ± 1.7 to 4.3 ± 1.7, corresponding to a 13% amelioration [13]. In addition, the reduction of mean FSS scores in both studies was >0.6, which has been suggested by Putzki et al. as a “clinical significant” improvement [27]. On the other hand, however, the final FSS score was still above 4.0, which is a widely accepted cut-off for fatigue [17]. Considering the high burden of MS-related fatigue, any alleviation would certainly be desirable, even if only marginal. However, by now we do not really know to what extent the observed reductions of the FSS score represent a benefit to affected patients, especially in terms of quality of life. Worthy of mention, a multivariate analysis in the study of Côté et al. failed to detect any improvement of quality of life after CPAP therapy, as examined by the Physical and Mental Component Summary Scale of the Short Form (36) Health Survey (SF-36) [13]. The number of studies on health-related quality of life in MS patients is very large, and fatigue has been identified as an independent predictor [28–30]. Thus, the lack of improvement in quality of life challenges the significance of CPAP therapy in MS-related fatigue. A possible explanation could be that the level of depression, which is an ever stronger predictor of quality of life in MS, did not improve during CPAP therapy, thereby obscuring a potential benefit [28–30]. 

 Additional considerations highlight the ambiguous relationship between fatigue and SDB in MS patients. First, we currently lack any objective tool that enables us to quantify fatigue. Thus, rating of treatment outcomes regarding fatigue relies solely on subjective estimation. Second, and closely related to the first point, a possible placebo effect cannot be ruled out. As evidenced by a recent randomized, double-blind controlled trial, the placebo effect of sham CPAP therapy may be quite considerable: ESS scores decreased in the placebo group from 15.2 ± 4.0 to 11.9 ± 5.9 (*P* = 0.001), corresponding to an effect size of 0.82, but results of the Osler test did not change [31]. Furthermore, the placebo group showed also a significant improvement of several items of the SF-36 [31]. Other groups confirmed this substantial placebo effect of CPAP therapy on sleepiness and quality of life [32]. A few groups studied fatigue response to CPAP therapy in SDB patients without underlying neurological disease, generating similar results [33–35]. While most groups uniformly reported a significant improvement of fatigue during CPAP therapy, the findings from 2 double-blind, randomized controlled trials observed significant reductions of fatigue scores also during placebo CPAP therapy [34, 35]. Recently, Tomfohr et al. confirmed that long-term CPAP therapy leads to a benefit on fatigue beyond placebo effects [18]. In summary, these studies clearly illustrate the importance of a randomized, placebo controlled trial in order to demonstrate that the observed improvement of fatigue in MS after CPAP therapy is more than a mere placebo effect.

 An intriguing finding of our study is the lack of correlation between sleepiness and SDB. Other groups made similar observations in MS patients, regarding both subjective and objective measures of sleepiness [12, 36]. In general, sleepiness is regarded as the main daytime consequence of SDB, caused by repeated apnea-induced arousals, sleep fragmentation, and reduced slow-wave sleep, and a marked reduction of sleepiness has been demonstrated following CPAP therapy [37]. The high prevalence of sleepiness in our cohort and its persistence under CPAP therapy suggest that SDB is not the main etiology of sleepiness in MS patients with fatigue. The prevalence of most sleep-wake disturbances in MS has been shown to be increased in MS compared to the general population and may thus account for the observed high prevalence of sleepiness [7]. On the other hand, sleepiness and fatigue in MS—generally regarded as two distinct symptoms—may share a common pathophysiology. The observation that the correlation between FSS and ESS scores in MS is much stronger than in subjects with various sleep disorders is also supportive of this hypothesis [38, 39].

 Finally, our study demonstrates that sleepiness, BMI, and disease severity are not useful predictors of SDB in MS patients with severe fatigue. Conversely, male and elder MS patients are more likely to present moderate-severe SDB. This illustrates that the presence of SDB among MS patients with severe fatigue may clinically not appear as obvious as it is in subjects with “classical” SDB. 

We have to acknowledge several limitations. First of all, the number of treated patients was small, making general conclusions difficult. Second, we did not assess possible confounders, such as mood disorders, and did not measure the effects of CPAP therapy on quality of life. Third, we did not include a control group. Furthermore, the additional presence of other sleep-wake disturbances has not been assessed. The prevalence of most sleep disorders is increased in MS patients [7], and effective improvement of fatigue in MS patients may require tailored therapies of more than only one sleep disorder [13, 14]. In particular, a recent meta-analysis indicated that restless-legs syndrome is frequent in MS, with reported prevalence of 12–58%, and restless-legs syndrome appears to be associated with fatigue in MS [40]. Finally, future studies should prefer polysomnography instead of respirography, as the latter does not allow detecting apnea-induced EEG arousals, and they might use additional fatigue scales such as the Modified Fatigue Impact Scale (MFIS), which has been shown to assess cognitive and psychosocial functioning more accurately than the FSS [41]. Nevertheless, we believe that our study provides an additional piece of clinical experience to the current discussion on the role of SDB in MS patients with fatigue.

In conclusion, our study confirms that SDB and fatigue are associated features in MS and that SDB treatment leads to a small yet statistically significant reduction of fatigue. However, well-designed studies are needed to substantiate the clinical relevance of this association, by proving that fatigue reduction following CPAP therapy has a meaningful impact on quality of life that goes beyond a mere placebo effect.

## Figures and Tables

**Table 1:** Demographic and clinical characteristics in severely fatigued MS patients with and without sleep-disordered breathing.

	No SDB (AHI < 5/h)	SDB (AHI ≥ 5/h)	*P *
*n* (%)	41 (59%)	28 (41%)	
Female sex, *n* (%)	32 (78%)	16 (57%)	0.06
Age (y)	47.4 ± 8.3	53.3 ± 9.5	**0.01**
BMI	25.4 ± 5.2	26.8 ± 4.2	0.27
Disease duration (y)	13.8 ± 8.9	13.6 ± 8.8	0.92
Disease subtype			0.73
RRMS	3	6	
SPMS	25	35	
EDSS	5.9 ± 1.5	5.8 ± 1.3	0.79
FSS	5.7 ± 0.7	5.5 ± 0.9	0.36
ESS	9.4 ± 4.7	9.7 ± 3.8	0.79
EDS (ESS ≥ 10)	18 (44%)	17 (61%)	0.13

BMI: body mass index (kg/m^2^), EDS: excessive daytime sleepiness, EDSS: Expanded Disability Status Scale, ESS: Epworth Sleepiness Scale, FSS: Fatigue Severity Scale, RRMS: relapsing-remitting multiple sclerosis, SPMS: secondary progressive multiple sclerosis.

**Table 2:** Gender-related differences in MS patients with severe fatigue.

	Female	Male	*P *
*n* (%)	48 (70%)	21 (30%)	
Age (y)	49 ± 9.3	51 ± 9.2	0.73
Disease duration (y)	14.4 ± 9.1	11.9 ± 7.7	0.16
Disease subtype			0.71
RRMS	7	2	
SPMS	41	19	
EDSS	5.9 ± 1.4	5.8 ± 1.4	0.78
ESS	9.7 ± 4.5	9.2 ± 3.8	0.65
EDS (ESS ≥ 10)	25 (52%)	10 (48%)	0.49
BMI	26.3 ± 5.2	25.3 ± 3.8	0.12
No SDB (AHI < 5/h)	32 (67%)	9 (43%)	0.06
AHI	5.2 ± 9.9	18.5 ± 24.7	**<0.001**
ODI	7.1 ± 7.9	18.1 ± 22.4	**<0.001**
SaO_2_ min (%)	78.9 ± 14.6	75.5 ± 19.1	0.58

AHI: apnea-hypopnea index, BMI: body mass index, EDS: excessive daytime sleepiness, EDSS: Extended Disability Status Scale, ESS: Epworth Sleepiness Scale, ODI: oxygen-desaturation index, RRMS: relapsing-remitting multiple sclerosis, SaO_2_ min: minimal O_2_ saturation, SDB: sleep-disordered breathing, SPMS: secondary progressive multiple sclerosis.
